# Risk of somatic diseases in offspring of survivors with childhood or adolescent central nervous system tumor in Sweden

**DOI:** 10.1002/ijc.33394

**Published:** 2020-11-23

**Authors:** Wuqing Huang, Kristina Sundquist, Jan Sundquist, Jianguang Ji

**Affiliations:** ^1^ Center for Primary Health Care Research Lund University/Region Skåne Malmö Sweden; ^2^ Department of Family Medicine and Community Health, Department of Population Health Science and Policy Icahn School of Medicine at Mount Sinai New York New York USA; ^3^ Center for Community‐based Healthcare Research and Education (CoHRE), Department of Functional Pathology, School of Medicine Shimane University Matsue Shimane Japan

**Keywords:** central nervous system tumor, epidemiology, offspring, somatic diseases

## Abstract

With the improvement of treatments, a growing number of survivors with childhood or adolescent central nervous system (CNS) tumor are parenting their own children. We aimed to explore the risk of somatic diseases among children of these survivors compared to population controls. Children of survivors with CNS tumor below age of 20 were identified between 1973 and 2014 by combining the several Swedish registers. Five children without parental CNS tumor were matched randomly to generate the population comparisons. Relative risk (RR) and absolute excess risk (AER) were calculated for overall somatic diseases, and hazard ratio (HR) was calculated for specific type of somatic diseases. A total of 2231 somatic disease diagnoses were identified in children of survivors with a cumulative incidence rate of 94.77 per 1000 person‐years, whereas the rate was 92.79 in matched comparisons thus resulting in an overall RR of 1.02 (95% CI = 0.98‐1.07) and AER of 1.98 (95% CI = −2.06, 6.13). Specifically, five of 1364 children of survivors had CNS tumor with an incidence rate of 0.21 per 1000 person‐year, whereas the rate was 0.04 in children of matched children, generating a HR of 4.91 (95% CI = 1.42‐16.96). Children of male survivors were at a statistically increased risk of malignancy, as well as infectious and parasitic diseases. In conclusion, no significantly higher risk of overall somatic diseases was found in children of survivors with CNS tumor before the age of 20, but children with a paternal diagnosis of CNS tumor had significantly increased risk of malignancies and infectious and parasitic diseases.

## Abstract 2

**What's new?**

Survivors of central nervous system (CNS) tumors are at higher risk of experiencing late adverse effects on reproductive function. However, it remains unknown if the tumor or related treatments subsequently affect the physical health of their offspring. This population‐based study did not find an association of parental CNS tutors in childhood or adolescence with the risk of overall somatic diseases in the offspring. Nonetheless, an increased risk was observed in preterm born children, and children of male survivors had an increased risk of malignancies and infectious and parasitic diseases, calling for a tailored surveillance strategy.

**Abbreviations**
AER: absolute excess risk
CI: confidence intervals
CNS: central nervous system
HR: hazard ratio
IR: incidence rates
RR: relative risk

## 1 INTRODUCTION

Central nervous system (CNS) tumor is the second most common cancer in the Swedish population below the age of 20 years.^1^ Due to the improvement in treatments, a growing number of patients diagnosed with CNS tumor survived for more than 5 years, with the rate in the 2010s being 75% for females and 61% for males in Sweden.^1^ Many of these survivors might plan to have children when they reach parenting age.^2^ Available evidence from population‐based studies suggested that cancer survivors experienced an increased risk of infertility and adverse birth outcomes; this indicates cancer itself or treatments play a role in the reproductive system.^3^, ^4^, ^5^, ^6^, ^7^, ^8^ Adverse birth outcomes have been linked to long‐term morbidity.^9^ Besides, damage in reproductive organs may lead to genetic or epigenetic mutations in gametes, which may subsequently influence the physical health of their children.^10^ Unfortunately, findings of the physical health in children of cancer survivors are limited and mixed.^11^, ^12^

We previously observed an elevated risk of being born preterm in children of survivors with CNS tumor before the age of 20 years by linking several Swedish nationwide registers.^13^ In this population‐based study, we further explored the somatic disease burden among offspring of these survivors by comparing the cumulative incidence rate of somatic diseases between children of survivors and the comparison children. Furthermore, we classified diagnoses of somatic diseases into 12 main diagnostic groups to provide a detailed assessment for specific somatic diseases and investigated the risk of specific type of disease among children of these survivors.

## 2 METHODS

### 2.1 Study population

All singleton live births between 1973 and 2014 were identified from the Swedish Medical Birth Register, which included all pregnancies that have led to childbirth in Sweden since 1973. We further identified parents of these children through the Swedish Multigeneration Register and obtained the information of CNS tumor diagnosis between 1958 and 2010 for parents from the Swedish Cancer Registry. We selected children whose parents were ever diagnosed with CNS tumor under the age of 20 years and their parents had survived for at least 5 years after the diagnosis. Five children, whose parents did not have a diagnosis of CNS tumor, were randomly matched to each child of survivors conditional on the same birth year (continuous), gender of offspring, maternal and paternal age at birth (continuous). To ensure that the child was conceived after parental diagnosis, children were excluded if they were born within 1 year after parental diagnosis with CNS tumor. Children with neonatal death were excluded if they died within 3 months after birth.

In Sweden, a unique individual national identification number is assigned to each resident living in Sweden longer than 3 months, which was replaced by a serial number to provide anonymity, and used to link several registers in our study.

### 2.2 Assessment of exposure and outcomes

Data about maternal or paternal diagnosis of CNS tumor, including date at diagnosis and histology of tumors, were retrieved from the Swedish Cancer Registry. CNS tumor was classified based on histology into the following: astrocytoma, neurinoma, ependymoma, meningioma, hemangioma and medulloblastoma. Other less common types and unknown histologic types were included in others.

Information on somatic diseases was collected from the National Patient Register (NPR); this register was created in 1964 and has data with completed inpatient care since 1987 and included patients treated in the specialized outpatient care since 2001.^14^ Each record in this register includes dates of admission, a primary diagnosis (the main reason for visiting a doctor) and a series of secondary diagnoses coded according to the International Classification of Disease (ICD), 7th version before 1969, 8th version between 1969 and 1986, 9th version between 1989 and 1996 and 10th version after 1996. The ICD‐7, ICD‐8 and ICD‐9 codes were translated to ICD‐10 codes to ensure consistency of the diagnosis during the study period. Only the primary diagnosis was included in the analyses. We classified the diagnoses into 12 main types of somatic diseases: infectious and parasitic disease (ICD‐10 codes: A00‐B99); malignant neoplasms (ICD‐10 codes: C00‐D09, D37‐D48); benign neoplasms (ICD‐10 codes: D10‐D36); disease of the blood and blood‐forming organs (ICD‐10 codes: D50‐D89); endocrine, nutritional and metabolic diseases (ICD‐10 codes: E00‐E90); diseases of the nervous system and sense organ (ICD‐10 codes: G00‐H95); diseases of the circulatory system (ICD‐10 codes: I00‐I99); diseases of the respiratory system (ICD‐10 codes: J00‐J99); diseases of the digestive system (ICD‐10 codes: K00‐K93); diseases of the skin and subcutaneous tissue (ICD‐10 codes: L00‐L99); diseases of the musculoskeletal system and connective tissue (ICD‐10 codes: M00‐M99); diseases of the genitourinary system (ICD‐10 codes: N00‐N99). If an individual was recorded more than once for a specific type of somatic diseases, only the first record was retained (ie, only the first incident diagnosis for somatic diseases was retained).

The primary outcome was the number of overall somatic diseases, which was calculated by summing the 12 main types mentioned earlier. The secondary outcome was a specific type of somatic diseases.

In addition, these children were further linked to the Cause of Death Register to identify the date of death and the cause of death.

### 2.3 Statistical analysis

Follow‐up for the subsequent somatic diseases commenced at the date of birth and ended at the date of death, date of emigration or end of the study (31 December 2015), whichever came first. The cumulative incidence rate of somatic diseases was calculated as the number of diseases divided by the person‐years of follow‐up. The risk ratio (RR) of somatic diseases was estimated by comparing the observed rate from children of survivors with the rate from the comparison children. The absolute excess rate (AER) was calculated as the difference of cumulative incidence rate between the study population and the matched comparisons. The 95% confidence intervals (CI) of the RR and AER were estimated using the methods described by Armitage and Berry.^15^ To explore the association in detail, stratified analyses were further performed based on the gender of child, year of childbirth (<2001 or ≥2001), maternal or paternal diagnosis, parental age at diagnosis with CNS tumor (childhood or adolescence), year of parental diagnosis (<1990 or ≥ 1990), first child after parental diagnosis or not, time interval between parental diagnosis and childbirth (1‐10, 11‐20 and ≥21 years), child being born preterm or not (gestational weeks <37 or ≥37), and histologic types of the parental tumor.

To investigate the risk of specific diagnostic groups, Cox proportional hazard model was used to calculate the hazard ratio (HR) and 95% CI. Follow‐up for diseases commenced at the date of birth and ended at the date of the first‐time record for the disease diagnosis, death, emigration or end of the study (31 December 2015), whichever came first. We further stratified the analyses by maternal or paternal diagnosis. Cox proportional hazard model was also used to explore the risk of a specific type of malignancy, in which follow‐up commenced at the date of birth and ended at the date of the first‐time record for any malignancy, death, emigration or end of the study (31 December 2015), whichever came first.

All analyses were performed using the SAS version 9.4 (SAS Institute, Cary, NC).

## 3 RESULTS

A total of 1364 children were born after parental diagnosis of CNS tumor, and 6820 children born from parents without CNS tumor were selected as the reference group matched by gender, year of birth, and maternal and paternal age at birth (Table 1).

**TABLE 1:** Sociodemographic characteristics among offspring of survivors with central nervous system tumor and matched comparisons

	Offspring of survivors		Matched comparisons	
Variables	Number of individuals	%	Number of individuals	%
Overall	1364	100	6820	100
Gender of offspring				
Female	633	46.4	3165	46.4
Male	731	53.6	3655	53.6
Year of childbirth				
<2001	721	52.9	3605	52.9
≥2001	643	47.1	3215	47.1
Maternal age at birth				
<25	304	22.3	1520	22.3
25 to 29	438	32.1	2190	32.1
≥30	622	45.6	3110	45.6
Paternal age at birth				
<30	530	38.8	2650	38.8
30 to 34	447	32.8	2235	32.8
≥35	387	28.4	1935	28.4

The association between parental diagnosis with CNS tumor and risk of somatic diseases is presented in Table 2. Children of survivors experienced a sum of 2231 diagnoses of the 12 types of somatic diseases, generating a cumulative incidence rate of 94.77 per 1000 person‐years, while the rate was 92.79 in the comparison children. When compared to the comparison children, children of survivors were not significantly associated with a higher relative risk of somatic diseases (RR = 1.02, 95% CI = 0.98‐1.07; AER = 1.98, 95% CI = −2.06, 6.13). No association was found in children of male survivors (RR = 1.05, 95% CI = 0.99‐1.13) or in children of female survivors (RR = 0.99, 95% CI = 0.93‐1.06). The association was statistically significant in children of survivors diagnosed during adolescence (RR = 1.11, 95% CI = 1.03‐1.19) rather than that during childhood. Further stratified analyses by preterm birth or not are listed in Table 3, showing that the positive association was observed in preterm‐born children (RR = 1.19, 95% CI = 1.01‐1.41) but not children born with full‐term (RR = 1.01, 95% CI = 0.96‐1.05), in particular, among preterm‐born children of female survivors (RR = 1.26) or childhood survivors (RR = 1.24). However, preterm birth played a small role in the association with paternal diagnosis or adolescent diagnosis. In addition, the RRs were negatively associated with the increase of time interval between parental diagnosis and childbirth, ranging from 1.05 in children born within 10 years since parental diagnosis, 1.01 in children born after 11‐20 years, to 0.95 in children born after more than 20 years since parental diagnosis.

**TABLE 2:** Relative risk and absolute excess risk of somatic diseases among offspring of survivors with central nervous system tumor compared to matched comparisons

	Number of outcomes		Number of person‐years		IR/per 1000 person‐years			
Variables	Offspring of survivors	Matched comparisons	Offspring of survivors	Matched comparisons	Offspring of survivors	Matched comparisons	RR (95% CI)	AER (95% CI)
Overall	2231	10 770	23 541	116 068	94.77	92.79	1.02 (0.98,1.07)	1.98 (−2.06,6.13)
Gender of offspring								
Female	1038	5155	10 692	52 915	97.08	97.42	1.00 (0.93, 1.07)	−0.34 (−6.37, 5.94)
Male	1193	5615	12 850	63 153	92.84	88.91	1.04 (0.98, 1.11)	3.93 (−1.46, 9.52)
Year of childbirth								
<2001	1373	6535	18 581	91 475	73.89	71.44	1.03 (0.98, 1.10)	2.45 (−1.59, 6.64)
≥2001	858	4235	4961	24 593	172.96	172.20	1.00 (0.93, 1.08)	0.75 (−10.58, 12.49)
Parental diagnosis								
Maternal	1181	5811	12 782	62 508	92.40	92.96	0.99 (0.93, 1.06)	−0.57 (−5.98, 5.05)
Paternal	1050	4959	10 760	53 560	97.58	92.59	1.05 (0.99, 1.13)	5.58 (−0.29, 11.69)
Parental age at diagnosis								
Childhood	1398	7010	15 366	75 212	90.98	93.20	0.98 (0.92, 1.03)	−2.22 (−7.14, 2.86)
Adolescence	833	3760	8175	40 856	101.90	92.03	1.11 (1.03, 1.19)	9.86 (2.89, 17.15)
Year of parental diagnosis								
<1990	2032	9661	22 386	110 191	90.77	87.67	1.04 (0.99, 1.09)	3.10 (−0.96, 7.27)
≥1990	199	1109	1156	5877	172.22	188.72	0.91 (0.78, 1.06)	−16.56 (−39.61, 8.30)
Time interval between parental diagnosis and childbirth								
1 to 10	1288	6099	13 302	66 089	96.83	92.28	1.05 (0.99, 1.11)	4.54 (−0.85, 10.13)
11 to 20	597	2911	6739	33 053	88.59	88.07	1.01 (0.92, 1.10)	0.52 (−6.73, 8.16)
≥21	346	1760	3501	16 926	98.84	103.98	0.95 (0.85, 1.07)	−5.15 (−15.7, 6.12)

Abbreviations: AER, absolute excess risk; CI, confidence intervals; CNS, central nervous system; IR, incidence rates; RR, relative risk.

**TABLE 3:** Relative risk and absolute excess risk of somatic diseases among offspring of survivors with central nervous system tumor compared to matched comparisons stratified by preterm birth or not

	Number of outcomes		Number of person‐years		IR/per 1000 person‐years		
Variables	Offspring of survivors	Matched comparisons	Offspring of survivors	Matched comparisons	Offspring of survivors	Matched comparisons	RR (95% CI)
Preterm birth							
Yes	186	563	1503	5427	123.78	103.73	1.19 (1.01, 1.41)
No	2045	10 207	22 039	110 641	92.79	92.25	1.01 (0.96, 1.05)
Parental diagnosis							
Maternal diagnosis							
Preterm	115	301	890	2935	129.29	102.57	1.26 (1.02, 1.56)
No preterm	1066	5510	11 892	59 573	89.64	92.49	0.97 (0.91, 1.03)
Paternal diagnosis							
Preterm	71	262	613	2493	115.79	105.10	1.10 (0.85, 1.43)
No preterm	979	4697	10 147	51 067	96.48	91.98	1.05 (0.98, 1.12)
Parental age at diagnosis							
Childhood							
Preterm	135	357	1080	3550	124.95	100.57	1.24 (1.02, 1.51)
No preterm	1263	6653	14 286	71 663	88.41	92.84	0.95 (0.90, 1.01)
Adolescence							
Preterm	51	206	422	1878	120.78	109.72	1.10 (0.81, 1.50)
No preterm	782	3554	7753	38 978	100.87	91.18	1.11 (1.02, 1.20)

Table 4 shows the RRs and AERs of somatic diseases in offspring of survivors diagnosed with a specific histologic type of CNS tumor. Children of survivors with ependymoma (RR = 1.16, 95% CI = 0.97‐1.38), meningioma (RR = 1.12, 95% CI = 0.96‐1.30) and neurinoma (RR = 1.12, 95% CI = 0.87‐1.46) were associated with a higher risk of developing somatic diseases, but none of them were statistically significant, which may be due to the limited number of cases.

**TABLE 4:** Relative risk and absolute excess risk of somatic diseases among offspring of survivors with central nervous system tumor compared to matched comparisons stratified by histologic type

	Number of outcomes		Number of person‐years		IR/per 1000 person‐years			
Variables	Offspring of survivors	Matched comparisons	Offspring of survivors	Matched comparisons	Offspring of survivors	Matched comparisons	RR (95% CI)	AER (95% CI)
Ependymoma	153	657	1534	7628	99.77	86.13	1.16 (0.97, 1.38)	13.61 (−1.84, 30.74)
Neurinoma	199	886	2234	11 110	89.08	79.75	1.12 (0.96, 1.30)	9.33 (−2.94, 22.78)
Meningioma	71	313	950	4711	74.74	66.43	1.12 (0.87, 1.46)	8.30 (−8.52, 27.98)
Hemangioma	72	351	626	3162	115.00	111.00	1.04 (0.80, 1.34)	4.01 (−21.38, 33.32)
Astrocytoma	1172	5930	12 675	62 302	92.47	95.18	0.97 (0.91, 1.03)	−2.72 (−8.16, 2.93)
Medulloblastoma	57	325	708	3455	80.52	94.07	0.86 (0.65, 1.13)	−13.56 (−34.11, 10.67)
Others	507	2308	4815	23 699	105.29	97.39	1.08 (0.98, 1.19)	7.91 (−1.29, 17.63)

Abbreviations: AER, absolute excess risk; CI, confidence intervals; CNS, central nervous system; IR, incidence rates; RR, relative risk.

**FIGURE 1:**
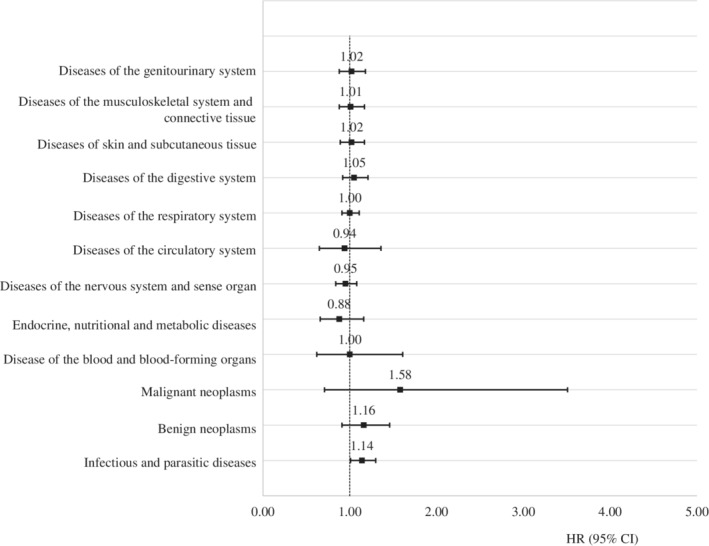
Hazard ratio of specific types of somatic diseases among offspring of survivors with central nervous system tumor compared to matched comparisons. CI, confidence intervals; HR, hazard ratio

**FIGURE 2:**
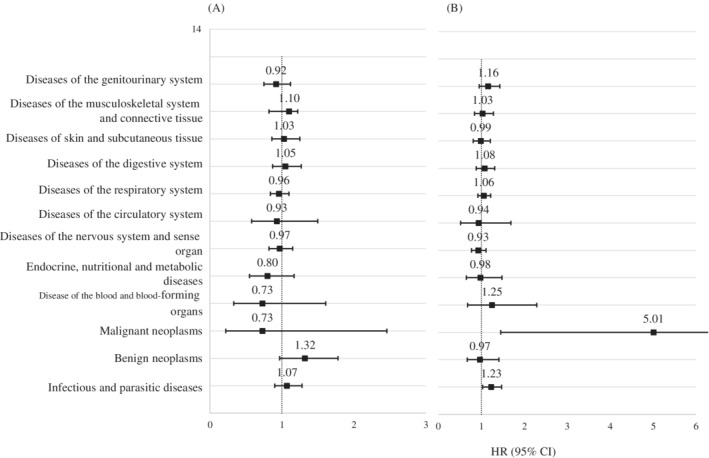
Hazard ratio of specific types of somatic diseases stratified by maternal or paternal diagnosis with central nervous system tumor. CI, confidence intervals; HR, hazard ratio. A, Maternal diagnosis; B, paternal diagnosis

The HRs and 95% CI for a specific type of somatic disease are shown in Figure 1 and Supplementary Table 1, and the stratified analysis by maternal or paternal diagnosis is shown in Figure 2 and Supplementary Table 2. Children of survivors were related to a 14% higher risk of being diagnosed with infectious and parasitic diseases than the corresponding children (95% CI = 1.01‐1.30), which was amplified in children of male survivors (HR = 1.23, 95% CI = 1.03‐1.47). Moreover, the highest HR was found for malignant neoplasm (HR = 1.58) and benign neoplasm (HR = 1.16) but they were not significant (Figure 1 **)**. However, as shown in Supplementary Table 3, children of survivors had a 4.91 times higher risk of CNS tumor (95% CI = 1.42‐16.96). Paternal diagnosis was mainly responsible for the increased risk of malignant neoplasm (HR = 5.01, 95% CI = 1.45‐17.3) (Figure 2). The incidence of other types of somatic diseases was comparable between children of survivors and their matched comparisons.

## 4 DISCUSSION

Findings from this population‐based study did not find an increased risk of overall somatic diseases in offspring of survivors diagnosed with CNS tumor below the age of 20 years when compared to the comparison children. An elevated risk was observed in preterm‐born children, especially in children of female survivors or childhood survivors. In terms of the pattern of disease burden, the offspring of male survivors experienced a greater risk of malignancy and infectious and parasitic diseases.

A previous cohort study conducted among the Danish population found that offspring of cancer survivors in childhood and adolescence were not associated with a higher risk of overall hospitalization with a HR of 1.05 (95% CI, 0.98‐1.12) when compared to the general population, but our study lacked detailed information for specific cancer type.^12^ Other two register‐based studies from Sweden demonstrated no difference concerning mortality between offspring of female or male cancer survivors compared to the general population.^16^, ^17^ In the present study, we also did not observe an increased risk of overall somatic diseases in offspring of survivors of childhood and adolescent CNS tumor. It is well‐known that preterm birth is an important factor regarding long‐term growth damage and morbidity.^18^ Our previous study found a significantly increased risk of preterm birth among offspring of female CNS tumor survivors.^13^ Several other population‐based studies also demonstrated an elevated incidence of experiencing preterm birth or other adverse pregnancy outcomes in female cancer survivors but not in male survivors.^3^, ^4^, ^5^, ^6^, ^7^, ^8^ After stratifying by preterm birth, we found that preterm birth strengthened the association of somatic disease risk with maternal diagnosis but played a small role in the association with paternal diagnosis. These findings might indicate that maternal diagnosis probably affects the physical health of their children via adverse birth outcomes, whereas paternal diagnosis might take an effect via genetic or epigenetic mutation. Emerging evidence suggested that epigenetic changes in male sperms could transfer to their children, which may contribute to disease susceptibility in offspring of mammals.^19^, ^20^, ^21^, ^22^, ^23^, ^24^, ^25^ For example, chemotherapy in male adolescents was found to alter sperm DNA methylation, which could be transmitted to the next generation and promote the epigenetic transgenerational inheritance of disease.^24^ Besides, age of parental diagnosis also modified the observed association with a higher risk in children of adolescent survivors than those of childhood survivors, but preterm‐born children of childhood survivors were at the highest risk.

Furthermore, the risk of somatic diseases became weaker in children of survivors diagnosed later than 1990, which may be related to the improvement of treatments or the development of in vitro fertilization. in vitro fertilization was first adopted in 1982 and was very rare before 1990 in Sweden.^26^ Existing data showed that the late effects of cancer treatment may last for years after cancer cure but declined with time since diagnosis.^27^, ^28^, ^29^, ^30^ In our study, the risk of somatic diseases declined with the increasing time interval between parental diagnosis and childbirth but none of them was statistically significant. Further study is needed to examine if survivors with CNS tumor should take into consideration of the timing being parents. Regarding stratified analysis by histological type of parental tumor, the statistical power was limited due to the small sample size in each stratum. Therefore, although no significant association was observed between any type of parental tumor and somatic diseases risk in children, the result should be interpreted with caution. This calls for further investigations.

In line with the previous studies for offspring of overall cancer survivors, regarding the pattern of somatic diseases burden, the highest risk was noted for malignant neoplasms with HR of 1.58, mainly for CNS tumor with HR of 4.91 in offspring of CNS tumor survivors.^11^, ^12^ Cancer susceptibility is heritable, and several malignant neoplasms were demonstrated to have familial aggregation, including nervous system tumor.^31^, ^32^, ^33^, ^34^, ^35^, ^36^, ^37^, ^38^ The causes of CNS tumors are largely unknown, but family history is an established risk factor although a large number of familial aggregations cannot be explained by specific genetic mutations.^37^ Our findings supported that parental CNS tumor was a risk factor for CNS tumor. Besides, the increased risk of overall malignant neoplasms was significant among offspring of male survivors rather than female survivors. The incidence rate of infectious and parasitic diseases was also significantly higher only in children of male survivors. As mentioned earlier, available evidence found that epigenetic changes in male sperms may lead to disease susceptibility in offspring of mammals, including cancer susceptibility.^21^ The mechanism behind the difference between maternal and paternal diagnoses is worth deep investigation.

This is, to the best of our knowledge, the first population‐based study to explore the physical health among children of survivors with CNS tumor in childhood or adolescence. The strengths of our study include the high quality and nationwide coverage of registers, the verified disease diagnoses and the large randomly selected matched comparisons. The main limitation is the lack of treatment data, making it unavailable to examine the impact of specific treatments on somatic health in offspring. Data about diagnosis in outpatients were not completed until 2001. However, the matched comparisons were selected conditional on the birth year to ensure the comparability of the cumulative incidence rate of somatic diseases between the two groups.

In conclusion, offspring of survivors with CNS tumor in childhood or adolescence were not associated with a higher risk of overall somatic diseases. But an increased risk was observed in preterm‐born children. Children whose father were ever diagnosed with CNS tumor in early life were related to an increased risk of malignancies, as well as infectious and parasitic diseases, which calls for a tailored surveillance strategy.

## CONFLICT OF INTEREST

The authors declare no conflict of interest.

## ETHICS STATEMENT

The Ethics Committee at Lund University approved (February 6, 2013) this nationwide cohort study (Dnr 2012/795). Written informed consent is not needed for the register‐based study in Sweden.

## Supporting information


**Supplementary Table 1** Hazard ratio of specific type of somatic diseases among offspring of survivors with central nervous system tumor compared with matched comparisons.
**Supplementary Table 2** Hazard ratio of specific type of somatic diseases among offspring of survivors with central nervous system tumor compared with matched comparisons, stratified by maternal or paternal diagnosis.
**Supplementary Table 3** Hazard ratio of specific malignant neoplasm among offspring of survivors with central nervous system tumor compared with matched comparisons.

## Data Availability

Data cannot be shared publicly due to the confidentiality under the Swedish legislation. Registry‐based data are available from the appropriate Swedish authorities (the Swedish National Board of Health and Welfare (https://www.socialstyrelsen.se/en) and Statistics Sweden (https://www.scb.se/en), for researchers who meet the criteria for access to confidential data.
