# Supplementary material for: Risk of somatic diseases in offspring of survivors with childhood or adolescent central nervous system tumor in Sweden
Source: Int J Cancer. 2020 Nov 23;148(9):2184–92. doi: 10.1002/ijc.33394 (PMC8048456; doi:10.1002/ijc.33394)
Supplement: Supplementary file 1 — Supplementary Table 1 Hazard ratio of specific type of somatic diseases among offspring of survivors with central nervous system tumor compared with matched comparisons. Supplementary Table 2 Hazard ratio of specific type of somatic diseases among offspring of survivors with central nervous system tumor compared with matched comparisons, stratified by maternal or paternal diagnosis. Supplementary Table 3 Hazard ratio of specific malignant neoplasm among offspring of survivors with central nervous system tumor compared with matched comparisons. [file IJC-148-2184-s001.pdf]

Risk of somatic diseases in offspring of survivors with childhood or adolescent central nervous system tumor in Sweden

Wuqing Huang, M.D. [1], Kristina Sundquist, M.D., Ph.D. [1,2,3], Jan Sundquist, M.D., Ph.D. [1,2,3], Jianguang Ji, M.D., Ph.D. [1]

Contents	Title
**Supplementary Table 1**	Hazard ratio of specific type of somatic diseases among offspring of survivors with central nervous system tumor compared with matched comparisons.
**Supplementary Table 2**	Hazard ratio of specific type of somatic diseases among offspring of survivors with central nervous system tumor compared with matched comparisons, stratified by maternal or paternal diagnosis.
**Supplementary Table 3**	Hazard ratio of specific malignant neoplasm among offspring of survivors with central nervous system tumor compared with matched comparisons.

Supplementary Table 1. Hazard ratio of specific type of somatic diseases among offspring of survivors with central nervous system tumor compared with matched comparisons.

Outcome	Number of outcomes		Number of person-years		IR / per 1000 person-years		HR(95%CI)
	Offspring of survivors	Matched comparisons	Offspring of survivors	Matched comparisons	Offspring of survivors	Matched comparisons	
**Specific disease**							
Infectious and parasitic diseases	301	1334	19860	101411	15.16	13.15	1.14(1.01,1.30)
Benign neoplasms	86	368	22973	113709	3.74	3.24	1.16(0.91,1.46)
Malignant neoplasms	8	25	23481	115864	0.34	0.22	1.58(0.71,3.51)
Disease of the blood and blood-forming organs and certain disorders involving the immune mechanism	20	99	23393	115138	0.85	0.86	1.00(0.62,1.61)
Endocrine, nutritional and metabolic diseases	59	331	23046	113390	2.56	2.92	0.88(0.66,1.16)
Diseases of the nervous system and sense organ	300	1554	20688	101385	14.50	15.33	0.95(0.84,1.08)
Diseases of the circulatory system	33	174	23301	114899	1.42	1.51	0.94(0.65,1.36)
Diseases of the respiratory system	490	2391	18864	90996	25.98	26.28	1.00(0.91,1.11)
Diseases of the digestive system	251	1181	21313	105678	11.78	11.18	1.05(0.92,1.21)
Diseases of skin and subcutaneous tissue	238	1158	21782	107693	10.93	10.75	1.02(0.89,1.17)
Diseases of the musculoskeletal system and connective tissue	219	1065	22041	108733	9.94	9.79	1.01(0.88,1.17)
Diseases of the genitourinary system	225	1087	21872	107952	10.29	10.07	1.02(0.88,1.18)

Abbreviations: CI, confidence intervals; HR, hazard ratio; IR, incidence rates.

Supplementary Table 2. Hazard ratio of specific type of somatic diseases among offspring of survivors with central nervous system tumor compared with matched comparisons, stratified by maternal or paternal diagnosis.

**Outcomes**	**Maternal diagnosis**	**Paternal diagnosis**
	**HR(95%CI)**	**HR(95%CI)**
**Specific disease**		
Infectious and parasitic disease	1.07(0.90,1.28)	1.23(1.03,1.47)
Benign neoplasms	1.32(0.97,1.78)	0.97(0.67,1.41)
Malignant neoplasms	0.73(0.22,2.46)	5.01(1.45,17.3)
Disease of the blood and blood-forming organs and certain disorders involving the immune mechanism	0.73(0.33,1.61)	1.25(0.68,2.29)
Endocrine, nutritional and metabolic diseases	0.80(0.55,1.17)	0.98(0.65,1.48)
Diseases of the nervous system and sense organ	0.97(0.82,1.15)	0.93(0.77,1.11)
Diseases of the circulatory system	0.93(0.58,1.50)	0.94(0.52,1.69)
Diseases of the respiratory system	0.96(0.84,1.10)	1.06(0.92,1.22)
Diseases of the digestive system	1.05(0.87,1.27)	1.08(0.88,1.31)
Diseases of skin and subcutaneous tissue	1.03(0.86,1.25)	0.99(0.81,1.21)
Diseases of the musculoskeletal system and connective tissue	1.10(0.82,1.22)	1.03(0.84,1.28)
Diseases of the genitourinary system	0.92(0.75,1.12)	1.16(0.95,1.43)

Abbreviations: CI, confidence intervals; HR, hazard ratio.

Supplementary Table 3. Hazard ratio of specific malignant neoplasm among offspring of survivors with central nervous system tumor compared with matched comparisons.

Outcome	Number of outcomes		IR / per 1000 person-years		HR(95%CI)
	Offspring of survivors	Matched comparisons	Offspring of survivors	Matched comparisons	
**Malignant neoplasms**	8	25	0.34	0.22	1.58(0.71,3.51)
**Specific malignant neoplasm**					
CNS	5	5	0.21	0.04	4.91(1.42,16.96)
Leukaemia	1	4	0.04	0.03	1.24(0.14,11.09)
Melanoma	1	2	0.04	0.02	2.47(0.22,27.19)
Cervix	1	1	0.04	0.01	4.93(0.91,78.85)
Testis	0	3	0.00	0.03	-
Colon	0	2	0.00	0.02	-
Connective tissue	0	2	0.00	0.02	-
Liver	0	1	0.00	0.01	-
Breast	0	1	0.00	0.01	-
Prostate	0	1	0.00	0.01	-
Kidney	0	1	0.00	0.01	-
Eye	0	1	0.00	0.01	-
Hodgkin’s disease	0	1	0.00	0.01	-

Abbreviations: CNS, central nervous system; CI, confidence intervals; HR, hazard ratio; IR, incidence rates.
